# A Cross-Sectional Study on Selected Correlates of High risk Sexual Behavior in Polish Migrants Resident in the United Kingdom

**DOI:** 10.3390/ijerph14040422

**Published:** 2017-04-14

**Authors:** Maria Ganczak, Grażyna Czubińska, Marcin Korzeń, Zbigniew Szych

**Affiliations:** 1 Department of Epidemiology and Management, Pomeranian Medical University, 70-204 Szczecin, Poland; 2 Department of Applied Psychology, Polish University Abroad, London W6 0RF, UK; grazyna.czubinska@puno.edu.pl; 3 Department of Methods of Artificial Intelligence and Applied Mathematics, West Pomeranian University of Technology, 71-210 Szczecin, Poland; mkorzen@wi.zut.edu.pl; 4 Department of Computer Science and Education Quality Research, Pomeranian Medical University, 70-204 Szczecin, Poland; zbigniew.szych@pum.edu.pl

**Keywords:** migrants, UK, risky sexual behavior, determinants, HIV infection

## Abstract

*Objective*: To assess the correlates of the high risk sexual behaviors of Polish migrants in the United Kingdom (UK) after 2004, and to compare such behaviors before/after immigration. *Methods*: In 2013, a cross-sectional study was conducted through the use of a *Computer-assisted web interviewing* surveying technique with the use of a self-administered questionnaire. *Results*: Among 408 respondents (56.9% women), with a median age of 32 years, significantly more admitted to having unprotected sexual contact with a casual partner while in the UK (*p* < 0.0001) than while in Poland; more were engaged in sex after the use of recreational drugs and alcohol (*p* < 0.0001 and *p* = 0.001 respectively). Being a male was associated with greater odds of unprotected sex, sex after the use of alcohol, and having multiple partners. Being single and having only been a resident for a short time in the UK, presenting a lower self-esteem, were predictors of unprotected sex. A total of 19.6% of the respondents admitted to having been tested while in Poland, a lower (*p* < 0.0001) frequency than while in the UK (49.5%); this referred to both genders; 1.2% (95% CI: 0.79–2.83%) reported that they were HIV positive. *Conclusions*: Migration can create a vulnerability to STIs, especially for single male migrants with low self-esteem, staying in the UK for less than two years. The results point to strengthening strategies which help reduce high risk sexual behavior among Polish migrants, and to introduce interventions to promote an awareness of HIV sero-status.

## 1. Introduction

In May 2004, ten more nations, including Poland, became members of the European Union (EU). This gradually gave citizens of these countries the opportunity to seek work in other EU member states, particularly those countries granting full work rights. Of note, central and east European nationals were allowed to work in the UK, Sweden, and Ireland immediately after accession. If the average length of stay abroad is considered, the UK became the most important destination country for Polish migrants, post EU enlargement [1]. Poland remains a top source of migrants in the UK, accounting for 14.9% of all foreign-born residents [2]. The migration of Poles to the UK seems significant in terms of the numbers of people involved [1]. These Polish post-accession migrants living in the UK are young, with a median age of 28 years; 81%–83% are aged from 20–34 years old [1,3].

The relocation to a new country initiates various cultural and psychological changes that involve a number of forms of mutual accommodation, leading to longer-term psychological and socio-cultural adaptations [4]. Such a process, in which heritage culture retention and English-culture acquisition represent separate dimensions, is defined within cultural studies as acculturation [5]. The adaptation to a new social environment might be particularly challenging when the migrants are settled in their new home, which contrasts culturally and socially to their homeland. The changes that occur can create cultural conflict and acculturative stress during intercultural interactions. They can also influence various aspects of the migrant’s health, including their sexual health. Although both are largely defined by social and economic factors [6], affecting many migrants at a higher rate, scant research has been carried out to identify the specific determinants of sexual health and the differences in vulnerability in different sub-populations of migrants. This gap in knowledge can be partly explained by the lack of consistent and comprehensive data [6].

While some recent research conducted in Europe [7,8,9], as well as in some other continents [10,11], has pointed to international migration as being one of the factors influencing risky sexual behaviors, the change in these behaviors at an individual level after migration has not been thoroughly quantified to a satisfactory level [12,13]. Gaining insight into the differences in sexually transmitted infections (STIs) risk behavior patterns between pre- and post-migration stages can provide a broader contextual understanding of how the shift from these two migration stages specifically impacts such behavior.

According to global literature, risky sexual behaviors among migrants are influenced by a wide range of personal, social, and cultural factors [12,13,14]. As an example, gender was reported to moderate the relationships between acculturation, and multiple partnerships and unsafe sex [14]. A younger age and the time spent in the host country were found to influence sex while under the influence of drugs or alcohol among Mexican migrants after migration to the U.S. [12]. Recent US Latino migrants with a lower education and higher incomes exhibited steeper decreases in pre- to post-immigration condom use [13]. It has also been proved that low self-esteem and poor satisfaction with life, experienced by some migrants, combined with the use of alcohol, led to risk taking and the loss of control over risk reduction strategies [15,16]. Understanding the factors which influence sexual health among Polish migrants may prevent serious consequences, such as STIs, and facilitate the formation of effective services for this group. The prevalence of STIs in the UK is one of the highest in the EU [17], and STIs continue to be a major public health problem, with 450,000 new STI diagnoses in 2013 and 100,000 people living with HIV. 

Although according to a study among central and eastern European (CEE) migrants to London [7], reports of prior STIs in this group were lower than in the general British population, 1.1% reported being HIV positive; this is a substantially higher prevalence than is estimated for British residents (0.09%). Of note, the majority (79%) of respondents from the CCE migrants study who reported being HIV positive were Polish by birth. Sadly, CEE patients made up a notable minority of patients attending genitourinary medicine clinics [18].

## 2. Objectives

More needs to be done to improve the understanding of the relationship between migration and sexual health, and to address migrant sexual health. Therefore, the objective of this study was to assess the correlates of high risk sexual behaviors of adult Polish migrants in the UK after 2004, and to compare such behaviors before and after immigration.

## 3. Materials and Methods

### 3.1. Population and Sampling

The cross-sectional study was conducted between March and August 2013 among recent post-accession Polish migrants in the UK. As there is no adequate sampling frame for this new migrant population [7,19] the study relied on convenience sampling. The survey was advertised with the collaboration of the Polish offline media (newspapers such as Cooltura, Londynek.net, Mojawyspa, Emito, and Polish Express) and online (through the web sites provided by the Polish Centre for Sexual Health, Polish Professionals in London, and London’s Polish University Abroad). The advertisement included a short description of the study and a short interview with one of the investigators on the study’s goal and objectives, together with a link to the dedicated website www.ankietka.pl (the largest Polish system for comprehensive survey studies) utilized for the purpose of this survey. The following eligibility criteria needed to be met in order to participate in the study: self-identification as a recent post-accession Polish migrant in the UK, aged 18 years or over, and agreement to take part in the study. Recruited participants with whom contact had already been made could use social networks to refer the web site to other people who could potentially participate in the study (i.e., a snow-ball sampling was used, with existing study subjects recruiting future subjects from among their acquaintances). To preserve the anonymity of the respondents, a Computer-Assisted Web Interview method was used. This is an Internet surveying technique in which the interviewer fills in questions following a script provided on the website. The research questionnaires are made in a program for creating web interviews and are shown on the site in such a way as to be available online for respondents to fill out [20].

### 3.2. Study Instrument 

A self-administered questionnaire was designed with the use of a literature review [7,8,9,12,13]. It contained 18 questions written in the Polish language that queried participants on the following:

1. socio-demographic: age; gender; education level (“primary school”, “high school”, “college”, “university”, “post-graduate”; for analysis, these options were dichotomized into “university and above level”, and “below university level”); marital status (“single”, “married”, “cohabitation”, “divorced”, “widowed”; for analysis, these options were dichotomized into “single” i.e., single/divorced/widowed and “non-single”, i.e., married/cohabitation); religion (“catholic”, “other religion”, “atheist”; for analysis, these options were dichotomized into “religious”, i.e., catholic/other religion and atheist); sexual orientation (“heterosexual”, “homosexual”, “bisexual”, “asexual”; for analysis, these options were dichotomized into “men who have sex with men (MSM)” and “other sexual orientation”); professional situation (“employed part-time”, “employed full-time”, “student”, “unemployed” and “retired”; for analysis, these options were dichotomized into “non-employed”, i.e., student/unemployed/ retired and “employed”, i.e., employed part-time/employed full-time); length of stay in the UK (in years: “≤2 years”, “3–5 years”, “6–8 years”, “≥8 years”; for analysis, these options were dichotomized into ≤2 years/>2 years); and place of residence in Poland (“town”, “village”);
2. risky sexual behavior in Poland in the past 12 months during the before-migration period and in the UK in the past 12 months (any unprotected sexual contact: with a casual partner; after the use of alcohol, i.e., having sex while feeling drunk; after the use of recreational drugs; or having multiple sexual partners).
3. determinants of risky sexual behaviors:
  personal: self-esteem measured by the Rosenberg self-esteem scale (RSES) [21].
  environmental/cultural: a. life satisfaction measured by the Berry’s MIRIPS Questionnaire [22], b. the history of sexual education at school (“yes”, “no”).
4. HIV testing; participants were asked to answer the following question: Have you ever been tested for HIV: in Poland/in the UK? (“yes”, “no”).
5. A previously diagnosed HIV infection; participants were asked to answer the following question: Have you had an HIV infection diagnosed by a medical professional? (“yes”, “no”).

Respondents were able to review and change their answers through a Back button. The survey tool was pilot-tested with 30 members of the immigrant community (results included). After reviewing the comments from the respondents included in the pilot study (which mainly referred to technical problems with the website, suggestions to add an open question/one more answer, or to write a more detailed clarification regarding certain questions), a few amendments were made to improve the clarity of the questions for the study population. Informed consent was obtained from all participants through the dedicated web site. The study received approval from the Ethical Committee of London’s Polish University Abroad (reference number PUNO-EC/1/2013).

### 3.3. Statistical Analysis 

Data were validated using a customized program STATISTICA PL Version 12.5. (StatSoft Inc., Tulsa, OK, USA, 2016) and R (R version 3.0.2) software [23]. Our main outcomes were risky sexual behaviors in the UK:

1. unprotected sexual contact with a casual partner,
2. sexual contact after the use of alcohol or recreational drugs,
3. having multiple sexual partners.
  in the context of:
  - the pre- and post- migration comparison (a McNemar’s test was utilized for assessing any significant differences between two correlated proportions, based on the same sample of subjects, i.e., risky sexual behavior in Poland and in the UK).
  - variables influencing these outcomes while in the UK.

A bivariate analysis assessed the demographic characteristics (age, gender, residency, education, marital status, employment, religion, and sexual orientation), together with the time spent in the UK and previous sexual education, associated with the outcome variables. The categorical variable groups were compared using the chi-square test with Yates correction and Fisher’s exact test; the Mann-Whitney test was used for the numerical variables. All of the variables significant (*p* < 0.05) at the bivariate level were used to build a multivariable logistic regression model (using a stepwise selection, and a backward procedure); with the help of R software [19]. The results are presented in Table 4. 

## 4. Results

Out of 1026 survey website visits, 500 individuals started the survey and 408 (82%) completed it.

### 4.1. Demographics

Among the 408 respondents (56.9% women), with a median age of 32 years, the vast majority (99.7%) were at least high school graduates, 74.5% came from Polish towns of between 50,000 and 500,000 inhabitants, most (89.2%) had regular employment in the UK, almost two thirds (63.9%) were married or living in an informal relationship (cohabiting), and 78.7% were religious (mostly Catholics). The vast majority of respondents (89.0%) were heterosexuals; there were 21 (5.1%) MSMs in the study. Almost half of the respondents (47.5%) came to the UK ≤2 years ago; Table 1.

### 4.2. Self-Esteem, Life Satisfaction, Sexual Education

More than one fourth of the respondents (29.0%) had a low self-esteem, measured with the use of RSES; 9.3%-average self-esteem and 61.5%-high self-esteem. A total of 18.4% of the participants had a low level of life satisfaction; 24.0%-an average and 57.6%-high. More than half of the respondents (53.2%) received regular sexual education at school. 

### 4.3. Risky Sexual Behaviours in Poland and in the UK 

Regarding unprotected sexual contact with a casual partner, 38.7% of the respondents admitted to having unprotected sexual contact with a casual partner while in the UK, a greater (*p* < 0.0001) frequency than while in Poland (20.6%); more were engaged in sexual contact after the use of recreational drugs in the UK than in Poland (10.0% vs. 2.2%; *p* < 0.0001), and also after alcohol intake (23.3% vs. 15.1%; *p* = 0.001); Table 2. A total of 27.9% of respondents admitted to having multiple sexual partners while in the UK, a lower frequency than while in Poland (31.1%); however, the difference was not statistically significant (*p* = 0.24). 

Males were significantly more often engaged in unprotected sex and sexual contact after the use of recreational drugs/alcohol than while in Poland (*p* < 0.0001, *p* = 0.02, *p* = 0.002 respectively), and less frequently reported having multiple partners (*p* = 0.008), while females were significantly more often engaged in sexual contact after the use of recreational drugs (*p* < 0.0001).

### 4.4. HIV Testing in Poland and in the UK 

Regarding HIV testing, 19.6% of the respondents admitted to having been tested while in Poland, a lower (*p* < 0.0001) frequency than while in the UK (49.5%); this referred to both genders (Table 3). Males significantly less often reported being tested for HIV while in Poland than females (14.2% vs. 23.7%; *p* = 0.02). Five participants (1.2% 95% CI: 0.79%–2.83%) reported that they were HIV positive. 

### 4.5. Variables Influencing Risky Sexual Behaviours While in the UK

Bivariate analyses of the determinants influencing risky sexual behaviors among Polish migrants in the UK are presented in Table 4. All variables significant at the bivariate level were then used to build a logistic regression model. Table 5 presents these models regarding the possible associations of various types of risky sexual behavior with selected variables. A male gender (OR = 3.55), being single (OR = 1.85), having low self-esteem (OR = 1.82), and a short duration in the UK (OR = 3.13), were each associated with a greater odds of unprotected sex with a casual partner. A male gender was also associated with a greater odds (OR = 2.50) of sexual contact after the use of alcohol, while a short time in the UK (OR = 4.62), having a job (OR = 8.44), and a higher education (OR = 3.23) were associated with a greater odds of sexual contact after the use of recreational drugs. A male gender, being single, and being an atheist were each associated with a greater odds of having multiple sexual partners (OR = 1.91; OR = 2.08; OR = 2.19 respectively). 

## 5. Discussion

### 5.1. Results Overview

Although some other studies assessed the sexual attitudes and lifestyles of central and eastern European CEE migrants in the UK in general [18,19], this is the first study to explore not only the risky sexual behavior among Polish migrants in the UK (which are the most numerous sub-population), but also the change in these behaviors after migration at an individual level. Overall, the results of this survey indicate high rates of risky sexual behaviors among Polish migrants in the UK, which are significantly more frequent than while in Poland, including unprotected sexual contact with a casual partner, and sexual contact under the influence of recreational drugs and alcohol. Being a male was associated with greater odds of unprotected sex with a casual partner, sex after the use of alcohol, and having multiple partners, while being single—with greater odds of unprotected sexual contact and promiscuity. Having been in the UK for a short period of time was associated with greater odds of unprotected sexual contact with a casual partner and sexual contact under the influence of alcohol. The reported HIV prevalence among Polish migrants was much higher than the estimated prevalence observed in the general Polish and British populations [24,25].

### 5.2. High Risk Sexual Behaviors before and after Migration 

Like other acculturating individuals, Polish migrants may face challenging experiences that can result in health disturbances, including mental and sexual health. Mental and sexual health is susceptible to the multifaceted interactions related to migration. There have been explanations for the vulnerability of migrants, such as long periods away from the social control of family and spouses, broader social and religious morals, social exclusion, limited access to information due to a poor language ability and/or legal status (for example, a lack of residence status and health insurance), and difficulties in the acculturation process [8,13]. Impaired mental health can lead to increased health risk behavior, such as alcohol and drug abuse [26,27]. Substance use was defined as a longitudinal predictor of risky sexual behavior [12,15,28,29] and was found to be significantly associated with an increased risk of the incidence of HIV infection [30]. Sex while under the influence of drugs was found to be prevalent among migrants, especially males [12]. 

Of note, the use of illicit psychoactive substances is not a minority activity amongst young people in the UK [31]. Relatively easy access to such substances in the UK and social acceptance might influence the reasons or motivations for their use regarding Polish migrants, and illustrate a passive reaction to the context in which a substance is available, rather than the deliberative decision to use a drug, based on a rational appraisal process [32]. According to the medical literature, the reported reasons regarding the decision to use a drug, vary from quite broad statements (e.g., to feel better) to more specific functions for use (e.g., to increase self-confidence) [31,32].

The aforementioned issues might explain the increase in the rates of risky sexual behaviors among Polish migrants in the UK when compared with rates before immigration. Furthermore, this new environment can give Polish migrants a sense of anonymity that facilitates risky behavior.

Similar to the results presented in this study, a survey focusing on recent Latino immigrants in the U.S. noted that migrant men were also significantly more likely to engage in sex while under the influence of drugs or alcohol after migration; a decrease in condom use among males was observed after migration to the U.S. [11]. We found differences in the risk behavior trajectories of males and females which corresponds with the findings of the U.S. study [12]. Polish men reported significant decreases in the number of sexual partners post immigration, while women reported increases in the number of sexual partners after immigrating to the UK.

### 5.3. Correlates of High Risk Sexual Behaviors after Migration

The results of this study show that numerous variables correlate with high risk sexual behaviors after migration to the UK. 

Du et al. reported that gender is a notable moderator of the relation between acculturation and sexual risk behavior [14]. Gender differences in sexual behaviors may be connected with migrants’ cultural values. As an example, traditional Latino cultures discourage sexual activity among teenage girls. During the acculturation process, Latino females may reject the traditional values on sexual abstinence and engage in risky sexual behaviors [33]. In contrast, Latino men may experience a small change in sexual behaviors as they are not restricted by the norm of sexual abstinence. Furthermore, acculturation may lead to less family support, so that more acculturated females are more likely to seek attachment in partner relationships and thereby get involved in risky sexual activity [34].

Indeed, sexual risk behaviors among Polish migrants in the UK differed across genders. While in Poland, significantly more men reported condom use than women, which is in line with another Polish study [35]. This can be related to gender power inequalities and the lack of a negotiating capacity to request safer sex, which renders women particularly vulnerable to a partner’s risky behavior [36]. However, after immigration to the UK, the trend has changed in both genders, i.e., condom use in males decreased to a high extent, from 82% to 46%, indicating a decreased adoption of this preventive method. Being a male was associated with a 3.5 times greater odds of unprotected sex with a casual partner. Declining condom use in males might be partly interpreted as an indicator of a broader availability of other means of contraception in the UK compared to Poland. Nevertheless, condom use did not decline in the women surveyed in this study. Additionally, a male gender was an independent predictor of sexual contact under the influence of alcohol and of having multiple sexual partners. This was also reported by other authors who conducted studies among migrants [7,16] and non-migrants [37,38]. As an example, in the study conducted among young adults in the US, the authors found that being male and single were significantly associated with having had five or more sexual partners in the past 12 months [38]. 

Being single was associated with about a two times greater odds of unprotected sexual contact and of having multiple partners in this study. Considering the fact that one third of the respondents were living alone, which reflects the migrant population data [1,7], this finding must be noted as of special interest in the context of the magnitude of the problem and the increased likelihood of potential STI acquisition from an infected partner(s).

Experience of migration may negatively influence self-esteem [26,32]. According to Panchanadeswaran et al., migrants lacking the resources to cope with negative events, as discrimination, may experience increased feelings of inadequacy and hopelessness, negative self-perceptions, resulting in low self-esteem [39]. As an example, several studies suggest that negatively experiencing discrimination affects self-esteem among Latino and Asian American migrant adolescents [40]. A number of authors suggest that low self-esteem, often connected with substance use, can lead to risky sexual behaviors [15,28,29]. For Polish migrants in the UK, this variable was a predictor of unprotected sexual contact. 

The results of this study show that migrants with a higher educational status and those employed were much more likely to be engaged in this type of behavior. One motivation for trying recreational drugs is the desire for novel experiences [41,42]. According to Wilmoth, more intelligent people tend to value novelty more highly and may therefore be more likely to have tried recreational drugs. Using data from the National Longitudinal Survey of Youth 1979, the author showed that intelligence tended to be positively related to the probabilities of having tried alcohol and several other recreational drugs [41]. Some other studies have also pointed towards higher education qualifications as a predictor of substance use [16].

A short period of time spent in the UK was found to be significantly associated with high risk sexual behavior in this study, such as unprotected sexual contact with a casual partner and sexual contact under the influence of recreational drugs. However, according to a recent study on sexual and HIV risk behavior in CEE migrants in London (41% of them from Poland), which took place between 2008 and 2009, no associations between time in the UK and the high risk sexual behaviors were found [7]. Regarding CEE MSMs, the longer that men had been in the UK, the more likely that they were to report recreational drug use in the past year and to have more than 10 male partners in the last five years [43]. A possible explanation of our findings could be that shortly after migration, Polish migrants (young and frequently single) have to deal with the long episodes living apart from their spouse or regular partner, and therefore may feel the accompanying need to look for a relationship to recompense the alienating aspects of the migration experience [8]. Of note, fewer social controls on behavior, as well as the experience of social isolation, which may be more expressed in the beginning of the stay in the UK, and a lack of experience of the British health system, may render migrants at risk regarding sexual health [18]. 

### 5.4. HIV among Polish Migrants in the UK

According to the study conducted by Evans et al. of CEE migrants in London, the reported HIV prevalence was similar (1.1%) to that observed by us (1.2%) [18,19]. Nevertheless, the lack of standardization in data collection makes it difficult to establish a clear picture of the burden of infectious diseases in migrant communities [6]. The overall reported HIV prevalence in our study was also similar to that observed in another survey carried out with samples of migrants from Spanish cities (1.7%) [8]. 

Migrants form eight CEE countries (Czech Republic, Estonia, Hungary, Latvia, Lithuania, Poland, Slovakia, and Slovenia) surveyed by Burns et al. [7] were much less likely (31%) to be tested for HIV (i.e., had ever had an HIV test) than our study participants (49.5%); both rates were much higher than that observed among the UK citizens in the Natal Survey of Sexual Attitudes and Lifestyles (Natsal) [44]. Hypothetically, the participants of our study might be those more interested in sexual health matters which might have had an impact on STI/HIV testing. Easy access to such testing in Poland may be another facilitator [24]; the data suggest that CEE migrants migrate for relatively short but recurring periods and continue to access healthcare in their home countries [7,18,25]. Of note, only one in five of our study participants reported having been tested for HIV while in Poland, which is a much lower (*p* < 0.0001) frequency than while in the UK; this referred to both genders. Potentially, migrants may be aware of their risky sexual behaviors in the UK, which are more frequent than while in Poland, and therefore utilize screening opportunities more often. 

Nevertheless, the reported HIV prevalence among Polish migrants is worrisome, especially in light of the low estimated prevalence observed in the general Polish [24] and British population [25].

## 6. Limitations

Firstly, the study design (cross-sectional) limits the ability to draw causal inferences. Secondly, a random sampling method was not used to select the study population. Therefore a self-selection bias was possible. However, it was difficult to create a proper sampling frame due to the scant information available on some immigrant sub-groups, such as undocumented or recent migrants [18]. Collecting data through the CAWI method, used in this survey, might have led to an over-representation of more wealthy migrants. Though, as internet access is universal in the UK, not only at home and work, but also through internet-cafes and mobile phones, we might be confident that the sampling procedure allowed for a representative sample of Polish migrants. Moreover, the participant socio-demographic characteristics are in line with data on the Polish immigrant population in the UK [1]. While we highlighted some variables regarding the patient demographic, other factors, e.g., sexual orientation, might have also influenced risky sexual behaviors. Because of the high refusal rate, possibly due to the sensitive and intimate nature of the study subject, the real prevalence of high risk behaviors among the study population could be under- or overestimated. Finally, because biological samples from the respondents were not obtained in this study, and the authors had to rely on self-reported respondent information, it can be hypothesized that the factual proportion of HIV-positive immigrants among the sample might be different.

## 7. Conclusions

These findings highlight the potential increasing risk for STIs of Polish immigrants in the UK who have frequent (more frequent than before immigration) unprotected sexual contact, sex under substance use, and sex with multiple sexual partners, but also if they acquire STIs, the increasing likelihood of transmitting such infections to their sexual partners. Furthermore, these results may explain, to some extent, how migration can create a vulnerability to STIs, especially for single male migrants with low self-esteem and a short duration of stay in the UK.

### Implications for Intervention

Such findings have implications for the targeting of prevention and intervention efforts for migrants tailored to facilitate the access to specialists to promote and maintain psychological and sexual health. Difficulties related to discussing intimate relations in a foreign language can prevent the uptake of services [18]. A lack of culturally sensitive information in a relevant language, suitably trained professionals, and services tailored to the specific needs of migrants may also be barriers. Policies, laws, and regulations governing service delivery can influence the access to and uptake of services. Effective sexual health education, including the continued promotion of condom use, especially among single male migrants, would prevent STI transmissions among the general population. Culturally adapted actions, considering a population’s characteristics—including lifestyles and beliefs—such as Polish migrant media and social media marketing campaigns, as well as actions sponsored by Polish NGOs (e.g., the Polish Sexual Health Center), to raise STI/HIV awareness and to promote awareness of personal status would be of value. In order to improve our understanding of the complex dynamics of STI risk and the infection rate, further research is needed, which would include biological samples from migrants.

## Figures and Tables

**Table 1:** Main characteristics of the participants (*n* = 408).

Variables	No of Participants	%
*Age*, years (Me)	32 (19–57)	
*Gender*		
Men	176	43.1
Women	232	56.9
*Place of residence in Poland*		
Town >500,000 inhabitants	52	12.7
Town 50–500,000 inhabitants	304	74.5
Rural area	52	12.7
*Educational level*		
Primary school	3	0.7
High school	125	30.6
College	106	26
University	133	32.6
Post-graduates	39	9.6
No data	2	0.5
*Marital status*		
Single	115	28.2
Married	144	35.2
Divorced	22	5.4
Widow/er	2	0.5
Informal relationship	117	28.7
Separated	5	1.2
Other	3	0.7
*Sexual orientation*		
Heterosexual	363	89.0
Homosexual	15	3.7
Bisexual	27	6.6
Asexual	3	0.7
*Religious status*		
Catholic	321	78.7
Atheist	87	23.1
*Time spent in UK*		
0–2 years	71	17.4
3–5 years	122	29.9
6–8 years	164	40.2
>8 years	51	12.5
*Knowledge of own HIV status*	174	
Yes	234	42.6
No		57.4
*Sexual education at school on regular basis*		
Yes	217	53.2
No	191	48.8

**Table 2:** Risky sexual behaviors among study participants in Poland and in the UK (*n* = 408).

Variables	Poland *n* (%)			UK *n* (%)			*p* **
	Total			Total			
	Males *n* = 176	Females *n* = 232	*p* *	Males *n* = 176	Females *n* = 232	*p*	
	*n* (%)	*n* (%)		*n* (%)	*n* (%)		
unprotected sexual contact with a casual partner	84 (20.6)			158 (38.7)			<0.0001
	32 (18.2)	52 (22.4)	<0.0001	99 (56.3)	59 (25.4)	<0.0001	M < 0.0001 F 0.29
sexual contact after the use of illegal drugs	9 (2.2)			41 (10.0)			<0.0001
	6 (3.4)	3 (1.3)	<0.0001	16 (9.1)	25 (10.7)	<0.001	M = 0.02 F < 0.0001
sexual contact after alcohol intake	62 (15.1)			95 (23.3)			0.001
	33 (18.8)	29 (12.5)	<0.0001	57 (32.4)	38 (16.3)	0.002	M 0.002 F 0.16
having 5 and more sexual partners	127 (31.1)			114 (27.9)			0.24
	85 (48.3)	42 (18.1)	<0.0001	65 (36.9)	49 (21.1)	<0.0001	M 0.008 F 0.39

* *p*—difference between males and females; ** *p*—difference between Poland and the UK.

**Table 3:** HIV testing among study participants in Poland and in the UK (*n* = 408).

Variables	Poland *n* (%)			UK *n* (%)			*p* **
	Total			Total			
	Males *n* = 176	Females *n* = 232	*p* *	Males *n* = 176	Females *n* = 232	*p*	
	*n* (%)	*n* (%)		*n* (%)	*n* (%)		
Had ever had HIV test	80 (19.6)			202 (49.5)			<0.0001
	25 (14.2)	55 (23.7)	0.02	94 (53.4)	106 (45.7)	0.15	M < 0.001 F < 0.001

* *p*—difference between males and females; ** *p*—difference between Poland and the UK.

**Table 4:** Bivariate analyses of determinants of risky sexual behaviors among Polish migrants in the UK (*n* = 408).

Variables		Type of Risky Sexual Behaviour															
		Unprotected Sex				Sex after the Use of Alcohol				Sex after the Use of Illegal Drugs				Multiple Sex Partners			
		*n*	*N*	%	*p*	*n*	*N*	%	*p*	*n*	*N*	%	*p*	*n*	*N*	%	*p*
Gender:	male	99	176	56.3	<0.0001	57	176	32.4	0.0002	16	176	9.1	0.58	65	176	36.9	0.0004
	female	59	232	25.4		38	232	16.4		25	232	10.8		49	232	21.1	
Age:	≤30 years	94	243	38.7	0.98	58	243	23.9	0.74	20	243	8.2	0.14	68	243	28.0	0.98
	>30 years	64	165	38.8		37	165	22.4		21	165	12.7		46	165	27.9	
Residence:	rural	22	52	42.3	0.57	11	52	21.2	0.70	7	52	13.5	0.38	20	52	38.5	0.07
	urban	136	356	25.4		84	356	23.6		34	356	9.6		94	356	26.4	
Marital status:	single	94	263	35.7	0.10	56	263	21.3	0.20	21	263	8.0	0.06	54	263	20.5	<0.0001
	non-single	64	145	44.1		39	145	26.9		20	145	13.8		60	145	41.4	
Sexual orientation:	MSM	19	21	90.5	<0.0001	10	21	47.6	0.01	7	21	33.3	0.001	7	21	33.3	0.38
	other	139	387	35.9		85	387	22.0		34	387	8.8		86	387	22.2	
Religiosity:	yes	127	321	39.6	0.50	73	321	22.7	0.62	36	321	11.2	0.13	87	321	27.1	0.47
	no	31	87	35.6		22	87	25.3		5	87	5.7		27	87	31.0	
Literacy:	university	60	130	46.2	0.04	40	130	30.8	0.01	22	130	16.9	0.002	33	130	25.4	0.43
	below	98	278	35.3		55	278	19.8		19	278	6.8		81	278	29.1	
Permanent Job:	yes	148	364	40.7	0.02	89	364	24.5	0.11	40	364	11.0	0.07	109	364	29.9	0.01
	no	10	44	22.7		6	44	13.6		1	44	2.3		5	44	11.4	
Time in UK:	≤2 years	52	194	26.8	0.008	31	194	16.0	0.88	8	194	4.1	0.03	44	194	22.7	0.8
	>2 years	33	214	15.4		32	214	15.0		1	214	0.5		51	214	23.8	
Self-esteem:	low/average	93	157	59.2	<0.0001	49	157	31.2	0.003	21	157	13.4	0.08	53	157	33.8	0.04
	high	65	251	25.9		46	251	18.3		20	251	8.0		61	251	24.3	
Life satisfaction:	low/av.	83	173	48.0	0.001	51	173	29.5	0.01	25	173	14.5	0.01	54	173	31.2	0.21
	high	75	235	31.9		44	235	18.7		16	235	6.8		60	235	25.5	
Sexual education:	yes	7	35	20.0	0.02	8	35	22.9	0.95	2	35		0.37	6	35	17.1	0.14
	no	151	373	40.5		87	373	23.3		39	373			108	373	29.0	

**Table 5:** Logistic regression model: association of risky sexual behavior with selected variables (OR estimates, significance of coefficients using Wald’s test; *n* = 408).

Variables		Type of Risky Behaviour							
		Unprotected Sexual Contact with a Casual Partner		Sex after the Use of Illegal Drugs		Sex after the Use of Alcohol		Multiple Sex Partners	
		OR	95% CI	OR	95% CI	OR	95% CI	OR	95% CI
Gender:	male	3.55 ***	1.68–4.25	0.50	0.23–1.05	2.50 ***	1.53–4.12	1.91 **	1.19–3.10
	female	1.00		1.00		1.00		1.00	
Time in the UK:	≤2 years	3.13 ***	2.26–5.53	4.62 ***	2.15–11.1	1.45	0.90–2.38	1.50	0.94–2.41
	>2 years	1.00		1.00		1.00		1.00	
Education:	university degree	1.49	0.91–2.38	3.23 ***	1.47–6.25	1.49	0.09–2.50	-	-
	below	1.00		1.00		1.00			
Marital status:	single	1.85 **	1.14–2.86	-	-	0.69	0.88–2.38	2.08 **	1.28–3.57
	non-single	1.00				1.00		1.00	
Sexual orientation:	MSM	-	-	-	-	-	-	1.79	0.81–4.35
	other							1.00	
Religious:	no	-	-	-	-	-	-	2.19 **	1.30–3.70
	yes							1.00	
Job:	permanent/part	-	-	8.44 *	1.6–157.9	-	-	1.94	0.94–3.91
	unemployed							1.00	
Self-esteem:	low/average	1.82 **	1.05–3.17	−1.00	-	0.61	0.99–2.70	-	-
	high	1.00				1.00			
Life satisfaction:	low/average	-	-	-	-	1.53	0.39–1.10	-	-
	high					1.00			
Sex education at school:	no	2.27	0.98–5.88	0.36	0.05–1.42	-	-	-	-
	yes	1.00		1.00					

* *p* < 0.05; ** *p* = 0.01; *** *p* < 0.001.
